# On the Current Connection and Relation Between Health Informatics and Social Informatics

**DOI:** 10.2196/40547

**Published:** 2022-09-28

**Authors:** Zdenek Smutny, Vasja Vehovar

**Affiliations:** 1 Faculty of Informatics and Statistics Prague University of Economics and Business Prague Czech Republic; 2 Faculty of Social Sciences University of Ljubljana Ljubljana Slovenia

**Keywords:** biomedical informatics, conceptual view, clinical informatics, international perspective, medical informatics

## Abstract

Scholars from the health and medical sciences have recently proposed the term social informatics (SI) as a new scientific subfield of health informatics (HI). However, SI is not a new academic concept; in fact, it has been continuously used in the social sciences and informatics since the 1970s. Although the dominant understanding of SI was established in the 1990s in the United States, a rich international perspective on SI has existed since the 1970s in other regions of the world. When that perspective is considered, the fields of understanding can be structured into 7 SI schools of thought. Against that conceptual background, this paper contributes to the discussion on the relationship between SI and HI, outlining possible perspectives of SI that are associated with health, medical, and clinical aspects. This paper argues against the multiplication and inconsistent appearance of the term SI when newly used in health and medical sciences. A more explicit name for the area that uses health and social data to advance individual and population health might be helpful to overcome this issue; giving an identity to this new field would help it to be understood more precisely and bring greater separation. This labeling could be fruitful for further segmentation of HI, which is rapidly expanding.

## Introduction

Social informatics (SI) refers to research activities related to the interaction between information and communication technology (ICT) and modern society. SI has been continuously defined and used in various regions and contexts since the early 1970s [1]. A broad international discourse has already been overviewed [2], where 7 (US, German, Russian, Norwegian, Japanese, Slovenian, and UK) regional SI schools of thought were identified. The US school, founded by the pioneering work of Rob Kling (1944-2003) [3], is globally dominant, although a detailed overview of the SI literature showed that it is steadily declining [4].

In addition to these contributions, Pantell et al [5] recently proposed a new and specific understanding of the term SI as a subfield of health informatics (HI), “focused on the application of information technologies to capture and apply social data in conjunction with health data to advance individual and population health” [5]. Shachak [6] challenged whether SI is an appropriately chosen label here, given its establishment in the US school of SI dating back to the mid-1990s [3,7,8]. This stimulated Pantell et al [9] to provide further arguments on why SI is a suitable proposed name. We would like to contribute to this discussion from a broader perspective than that specific to the US school of SI [6] by introducing the international and multidisciplinary aspects of SI. This paper is intended for the health community. It, therefore, presents the detailed conceptual basis of SI, which is not as well-known in this community as the conceptual basis of HI [10].

## Development and Perspectives of Social Informatics

When considering the interaction between ICT and society, SI addresses the social aspects of ICT as well as ICT aspects of social issues, including ICT applications in social sciences [2]. However, there is no unified general understanding of this interaction, and numerous well-established research fields overlap and compete here: computational social science, information systems, information society, science and technology studies, ICT and society, internet studies, information sociology, computer-mediated communication, human-computer interaction, and many others. There are also 2 main perspectives in SI, which differ substantially. The first is a regional perspective, which is linked to the theoretical and conceptual backgrounds of the 7 SI schools [11,12]. The second is a broad international perspective, which encompasses the relevant thematic areas in which the self-declared term SI can appear with reference to academic and scientific discourse [2,13].

The research that declares itself SI covers only a portion of this interaction, and it is spread across various thematic areas. These thematic areas where SI can appear are broad but can be classified into 3 streams according to their scientific origin [2,14]:

1. *Social science*, where the social aspects of ICT can be observed at a personal, organizational, or societal level.
2. *Informatics* (including computer science, information science, and information systems), where ICT is applied in the social area, including the provision of public or business services. This stream also includes computer modeling; artifact or solution design; and the structuring, conceptualization, and processing of information (eg, information architecture, information visualization, or information design).
3. *Social science methodology*, where ICT can be involved in various stages of the social science research process: data collection, processing, and analysis. The ICT applications are understood here as tools for social science research.

The above scope of SI is thus much broader than the domain-specific application of informatics (stream 2) in social sciences (eg, business informatics, political science informatics, music informatics, and legal informatics) and broader than the sole social science perspective on SI (stream 1). The latter is actually one of the main focuses of the US school of SI, which concerns “the interdisciplinary study of the design, uses and consequences of information technologies that takes into account their interaction with institutional and cultural contexts” [3]. The US school of SI addresses the social aspects of ICT, as well as ICT aspects of social issues: “SI refers to the study of social aspects of computerization, including the role of information technology in social and organizational change, the use of information technologies in social contexts, and the way that the social organization of information technologies is influenced by social forces and social practices” [15]. Contextual grasping is typical for SI (not only in the United States) and can include cultural, institutional, social, ethical, legal, or other issues. SI is defined thematically and not methodologically [16], so SI is open to the use of different research methods. Recent review papers [2,4,13] have confirmed that the prevailing scientific contributions in SI, referring to any of the SI schools, remain conceptually strongly rooted in the social sciences (stream 1), despite the declining dominance of the US school.

Since the 1970s [1,14,17], numerous scholars have thus used the term SI for their research, addressing the interaction between ICT and society. Given the above-described broadness of the potential SI scope, it is not surprising that considerable differences exist in the academic communities between various regions or schools of SI. However, the introduction of a different understanding of scientific terms is not a new phenomenon. A typical example is already the term *informatics*, dating back to the 1950s and especially the 1960s. Currently, the most widespread understanding of informatics is the conception originating in France [18-21], which recognizes informatics as a broad computer-oriented discipline that covers information and computational processes, including the logical construction of computer systems. Due to historical circumstances, a different understanding of informatics evolved in the United States [22-24] because of the strong existence of previously established names of computer-oriented disciplines, such as computer science, computer engineering, information systems, or library and information science. The US understanding of informatics is thus referred to more narrowly as a specific computer application–oriented domain with a user-centered perspective [25] and primary rooted in (library and) information science. The main emphasis is on the nonnumerical use of computers, and the central point of interest is information and its processing [26].

Within this context, the history of the term *medical informatics* in the United States is also informative for the understanding of informatics in the United States. Thanks to an action of the International Federation for Information Processing (Technical Committee 4 on medical informatics), the term was promoted and later adopted in the United States in the 1970s [27-29]. The term medical informatics thus existed independently in the United States for many years without referring to the general notion of informatics or the existence of other domain-applied informatics—that is, before the stand-alone term “informatics” was generally accepted in the United States.

It should be added that according to Hersh [26], in the United States, the following terms are subsumed under the broad umbrella *biomedical and health informatics* (sometimes only components are used, such as *biomedical informatics* or *HI*): *bioinformatics* (cellular and molecular level), *medical* and *clinical informatics* (personal level), and *public health informatics* (population level). Relevant SI research may appear from the international view of SI and the perspective of Pantell et al [5] in areas of medical (and clinical) informatics and population health informatics.

Nevertheless, as presented in Table 1, all existing schools of SI, including the US one, agree that SI relates to the general interaction (or combination) of society with ICTs [2], the computerization of human activities [8], or the social aspects of computerization [3], where the term “social” relates to social sciences in general.

**Table 1:** Main thematic focus of 7 SI schools. Based on and modified from Smutny and Vehovar [2].

SI^a^ school	Origin in existing disciplines	Typical research focus	Basic sources
German school	Interdisciplinary informatics with sociology, economics, psychology, and social work and services	Interactions of software with individuals, organizations, and society; the use of information and communication technology in social work	Sozioinformatik: Wulf et al, 2018 [30]; and Sozialinformatik: Kreidenweis [31], 2012
Japanese school	Informatics, media, and communication studies	Study of social information, communication, and information processes in an information society	Kurosu [32], 2010
Norwegian school	Sociology, psychology, and sociotechnical research	Study of the design, deployment, and evaluation of information and knowledge systems	Maliţa [33], 2006; and Bråten [34], 1983
Russian school	Initially, library science and communication studies; currently, informatics, sustainable development, and the philosophy of information, education, and knowledge management	Interaction between society and ICT^b^, along with sustainable development, with strong educational and philosophical overlaps	Chugunov [35], 2012; Kolin [17], 2021; and Melnikova and Romanovskaya [36], 2021
Slovenian school	Sociology, statistics, and informatics	Use of ICT to study the interactions between ICT and society	Petric and Atanasova [37], 2013; and Vehovar [38], 2006
UK school	Sociology, information science, and science and technology studies	Transdisciplinary study of sociotechnical interactions	Davenport [39], 2008
US school	Information science, information systems, sociology, and computer science	Study of the design, uses, and consequences of information technologies in institutional and cultural contexts	Kling [3], 2007; and Fichman et al [7], 2015

^a^SI: social informatics.

^b^ICT: information and communication technology.

However, we should also note that various language-specific exceptions exist here. For example, the German understanding of SI, according to the German term “sozialinformatik” [40], focuses on the application of ICTs in social work and services, as the term “sozial” in German has a strong connotation with social work. Nevertheless, this is, in a large part, a specific aspect of some Germanic languages. Therefore, in Scandinavia [41,42] and Germany, SI sometimes focuses on the social, educational, and health sectors [40,43], although this is also not a general or prevailing understanding of SI in these countries. We may add that the notion of SI, in general, frequently suffers from various translation issues, arising from the ambiguity and vagueness of the notions of “informatics” and “social,” which are both already problematic (ie, they have multiple meanings) in English. Therefore, SI is sometimes (back)-translated to English as socioinformatics (Germany), socio-information studies (Japan), or social information science (China) [2,14,44].

In addition, the term SI is increasingly used as a self-declared general label in the second stream of SI, particularly in connection to so-called computational social science, where ICTs are applied in various social science domains [4]. The related occurrence of the SI term in databases and searches (Web of Science [WoS], Scopus, and Google Search) and its usage to label various activities associated with the interaction between the ICT and society are growing exponentially, much faster than scholarly literature on SI referring to any of the SI schools [4]. This expansion of the usage of the term SI is outside of any SI school; it is actually very surprising, and in a way, also problematic, because it stems from the superficial usage of the term SI and has no conceptual grounding and thus is hard to explain.

We can thus summarize that SI is a very specific attempt to encompass the research related to the interaction between ICT and society. For various reasons, SI did not gain general popularity, in part because of the numerous alternative scientific fields that also address this interaction, and in part due to language issues, but also due to its internal diversity across 7 schools of SI, which span well beyond the US school. The recent expansion of the usage of the term SI outside scholarly production and outside any conceptual grounding makes this scenario even more problematic. Still, SI is a well-articulated scientific field, which addresses the general (ie, not field-specific) scope of the interaction between ICT and society.

## Health Informatics and Social Informatics

The alternative definition of SI proposed by Pantell et al [5] focuses only on a specific health-oriented community—for instance, in their argumentation, they used only the MEDLINE database for the search [9]—thus being defined as a subfield of HI. Nevertheless, in principle, the situation can be conceptually reversed from the broadest international perspective of SI, where HI can be subsumed under SI [2] as a domain-specific application of ICTs in society (stream 2).

To present the broad international and multidisciplinary view of SI and its connection with HI, we referred to the world’s most recognized citation databases, WoS and Scopus. Notably, there is a limitation in that they only focus on English terms, and documents in regional languages are omitted (eg, eLibrary.ru [Russia] and J-Stage [Japan]).

In the following comparison, we focused on documents located in the WoS and Scopus citation databases, where a search term appears in the title, abstract, or keywords. The search used the following terms: (TITLE-ABS-KEY (“social informatics”) AND NOT TITLE-ABS-KEY (“Institute for Computer Sciences, Social Informatics”)); (TITLE-ABS-KEY (“health informatics”)); “social informatics” (Topic) not “Institute for Computer Sciences, Social Informatics” (Topic); and “health informatics” (Topic). We removed articles that only mentioned *Lecture Notes of the Institute Computer Sciences, Social Informatics, and Telecommunications Engineering* in the abstract, without a connection to SI. The results for the last decade are presented in Table 2.

**Table 2:** Comparison of search terms in the Web of Science and Scopus citation databases during 2012-2021 (source: Web of Science and Scopus search on February 8, 2022).

Year	Web of Science (documents), n		Scopus (documents), n	
	Health informatics	Social informatics	Health informatics	Social informatics
2012	169	7	262	12
2013	203	8	286	19
2014	171	8	283	24
2015	233	3	346	13
2016	228	7	335	11
2017	270	3	422	7
2018	256	12	382	14
2019	354	19	508	26
2020	385	6	545	15
2021	410	10	612	12
Overall	2679	83	3981	153

Here, the number of documents in the citation databases (WoS and Scopus) is given in parentheses. The number of documents in the last decade comprises 83 (WoS) and 153 (Scopus) SI documents and 2679 (WoS) and 3981 (Scopus) HI documents. The situation in terms of the total number of documents found (not only in the last decade) is similar, confirming that the SI output (WoS: n=210; Scopus: n=325) is very small compared to HI (WoS: n=3870; Scopus: n=6387). If we extend the search to nearby HI areas, such as medical informatics and clinical informatics, the number of documents increases (WoS: n=8894; Scopus: n=38,288).

From the total number of documents (WoS: n=210; Scopus: n=325) with SI in the title, abstract, or keywords, 16 (WoS) and 32 (Scopus) of these documents also refer to the following terms: health, medical, or clinical informatics. This finding implies that from an international perspective, SI research (stream 2), in part, overlaps thematically with HI and related fields. More evidence that SI research appears in the health domain can be found in Cech [45] and Hoeffner [46]. For example, Cech [45] focused on SI research and educational institutions and identified 5 main themes of SI from an international view, and 1 of them was eHealth. We should also add that throughout the review of all scientific publications that explicitly refer to SI [2,4,14], an entry that explicitly refers to SI as a subfield of HI was not found.

Similarly, the number of documents explicitly referring to SI in the MEDLINE database is almost negligible (6 papers), as stated by Pantell et al [9], in addition to 58 papers where SI is mentioned only through an affiliation. Notably, these 58 affiliations are likely referring to the standard SI understanding (and not to that in the context of the proposed HI subfield).

We can conclude that currently, roughly around one-tenth of SI scholarly publications refer to the areas related to HI, whereas, for now, the scholarly literature on HI rarely mentions the notion of SI (although it somehow often refers to SI through an affiliation, which is related to the standard SI understanding; ie, the general interaction between ICT and society, which is not narrowed to HI).

## Discussion

Various conceptually focused scholarly pieces of literature exist on SI [2,4,7,13]. Correspondingly, 7 SI schools of thought can be identified, with the US school (founded by Rob Kling and his colleagues in the 1990s) being the most globally visible, although its impact is steadily declining. However, the US school of SI, which is in large rooted in social sciences (stream 1)—and can be, with some simplification, denoted as a study of the social aspects of computerization—is constantly evolving [7,47], and scholars are adapting to current research trends relevant to other areas as well (eg, computational social sciences and sociotechnical systems research).

From an international and interdisciplinary perspective, one can argue that a certain commonly established concept, such as SI, cannot be replicated in a very specific academic community—especially in socioconstructivist scientific disciplines, such as informatics—without soundly elaborating on the relations to other scientific fields. The multidisciplinary nature of modern science suggests that at least minimal consistency must be preserved across disciplines. The existing definitions of informatics and SI sometimes vary, which is already confusing. However, they still address the interaction between ICT and society in general, although they stem from different historical, ideological, and methodological circumstances or language-specific aspects. Therefore, to avoid further (and radical) increases in inconsistencies—which would be, in this specific case, produced with full awareness and intention—we suggest that for the new SI as a subfield of HI [5], at least an elementary conceptualization must be made with reference to the existing international perspective associated with the established notion of SI. This conceptualization would increase the precision of the term and decrease confusion, particularly if the new term itself is already reflected in the corresponding hierarchy.

Consequently, due to the complexity and specifics of this newly proposed field [5,9], perhaps 3 or more words would be needed. Within this context, besides the terms *social determinants of health informatics* and *clinical social informatics*, which were already considered but rejected because of awkwardness [5], the potential combinations that could perhaps be considered include *health social informatics*, *social informatics in health*, *social health informatics*, *medical social informatics*, or *social informatics in medicine*. In particular, *health social informatics* astutely refers to the health and social perspectives’ connection.

We may add that the usage of a 3-keyword description (eg, *health social informatics*) is not only an unambiguous solution to denote a complex and specific field but also reflect similar practice in many other areas where nuances and precision are needed. An excellent example is the established notion of *public health informatics* [48], as well as the more newly proposed neighboring notion of *population health informatics* suggested by Kharrazi et al [49]. The latter is more general than public health informatics and overlaps with other informatics fields, such as public health informatics, clinical informatics, and consumer health informatics.

When addressing the fact that the term SI is already in use with different meanings, Pantell et al [9] justified their proposal by arguing that some terms often have more than 1 meaning anyway, stating the example of *medical sociology,* which covers *sociology of medicine* and *sociology in medicine*. However, this example [9] cannot be used in this situation, where an already established and well-elaborated term (SI) is replicated in another context and given an entirely different meaning. The stated example of medical sociology would be relevant only if the authors, for example, discussed whether SI encompasses *sociology of informatics* and *sociology in informatics,* or *social sciences of informatics* and *social sciences in informatics*, but not for the case where specific domain-applied informatics (from stream 2) declares its SI component as a stand-alone notion of SI. The existence of multiple meanings of SI in 1 region, such as the United States, will not only confuse scholars at the regional level (followers of Kling [3] and followers of Pantell et al [9]) but this confusion will be transferred to the international level as English is the primary language in scientific publishing.

The case of SI is particularly critical because almost all scientific disciplines have certain informatics components and certain social science components. Using the general notion of SI to denote the narrow contexts of components within a certain discipline (ie, HI) would clearly lead to multiplication and inconsistent appearance of the term SI across disciplines; it would also increase the assumption that scientific disciplines are entirely independent and isolated. Most of all, this notion would neglect the existing, established, and generally accepted broader notion of SI. With such an approach, for example, *music informatics*, one of many domain-specific applications of informatics (stream 2), may also directly use the term SI as its subfield when “information technologies are used to capture and apply social data in conjunction to music data.”

Beyond this notion, Pantell et al [9] justified their proposal by referring to how, currently, the term SI is rarely used in the PubMed database. However, there are 67 publications already using this notion, mostly as an affiliation with the general notion of SI, which is not a negligible number. With the proposed usage of SI as a subfield of HI, the scholars addressing social determinants of HI will increasingly encounter SI publications from social sciences and informatics, which are related to the existing (general) notion of SI (particularly streams 1 and 2) and vice versa. This usage will create unnecessary confusion and inconsistency within and across disciplines. Similarly, the SI affiliations will lose clarity, because the new usage of the term SI will mostly contradict the SI affiliations, which typically refer to the established meaning. It will also become increasingly ambiguous as to which meaning of SI is to be understood by an SI affiliation.

We should also warn that the US school of SI, which Pantell et al [5,9] and Shachak [6] exclusively refer to, is not the only research stream where SI appears. It is true that for various reasons, it still dominates in a number of scholarly publications, but its role is steadily declining [4]. In any case, in scholarly literature, the stand-alone notion of SI already has various established regional meanings, which basically all address the general interaction between ICT and society, not just a particular domain-specific application of informatics. In addition, we should also beware of the exponential usage of the term SI outside conceptually grounded scholarly literature, which may further increase confusion if another usage of SI appears.

A more explicit and descriptive name—we already identified some potential 3-word options above—could avoid all these problems, bringing much more separation and precision, without any awkwardness or losses when denoting a new discipline that focuses “on the application of information technologies to capture and apply social data in conjunction with health data to advance individual and population health” [5]. Such labeling could be highly fruitful also for further segmentation of HI, which is rapidly expanding and needs more structuring in subdisciplines, together with the corresponding labeling.

## Abbreviations

HI: health informatics
ICT: information and communication technology
SI: social informatics
WoS: Web of Science
